# TRANSMISSION DYNAMICS OF EBOLA VIRUS DISEASE WITH VACCINE, CONDOM USE, QUARANTINE, ISOLATION AND TREATMENT DRUG

**DOI:** 10.21010/ajid.v15i1.2

**Published:** 2020-12-14

**Authors:** Ahman Queeneth Ojoma, Omale Davidb, Asogwa Christopher Chukwumad, Nnaji Daniel Ugochukwue, Mbah Godwin Christopher Ezikek

**Affiliations:** * Department of Mathematics, University of Nigeria, Nsukka; ** Department of Mathematical Sciences, Kogi State University, Anyigba, Kogi, Nigeria

**Keywords:** Ebola Virus Disease, EVD – Free Equilibrium, EVD – Endemic Equilibrium, Bifurcation Analysis

## Abstract

**Background:**

Ebola Virus Disease (EVD) has brought the human population, especially the West African race, great losses in so many areas such as economic productivity and human life. During the 2014 Ebola Virus outbreak, the disease devastated and threatened the whole world. EVD symptoms (fever, diarrhea, vomiting, etc) may appear anywhere between two to twenty-one days after infection. Those that recovered from the disease return to being susceptible again and can transmit the virus through semen as research has shown the virus presence in semen even after recovery.

**Material and Methods:**

Mathematical modeling method with the combination of vaccine, condom use, quarantine, isolation and treatment drug together as control measures in a population consisting of human and animals. A model system of non-linear differential equations for the control of EVD was formulated and the model effective reproduction number (*R_E_*) was obtained using the next generation matrix method and used in the stability analysis of the model. Center manifold theorem was used in the bifurcation analysis of the model.

**Results:**

The result shows that the stability analysis of the model shows that the EVD – Free Equilibrium is locally asymptotically stable when *R_E_* > 1 and EVD - Endemic Equilibrium is locally asymptotically stable when *R_E_* > 1. The model was shown to exhibit a forward bifurcation.

**Conclusions:**

Numerical simulations and analysis of the model show that EVD could be effectively controlled and eradicated within a short period of time when vaccine, condom use, quarantine, isolation and treatment drug control measures are implemented together.

## Introduction

Ebola Virus Disease (EVD), which was also known as Ebola hemorrhagic fever, is an uncommon and a very deadly disease caused by one of its five known types called Zaire Ebola Virus (EBOV). It can cause disease in human and non-human primates (Washington State Department of Health, 2018). Ebola virus was first discovered in 1976 near the Ebola River in what is now known as Democratic Republic of Congo, since then there have been several outbreaks in Africa (Rivers *et al.*, 2014). The 2014 Ebola outbreak in West Africa is related to EBOV virus. It was the most widespread in the history of the disease with so many countries affected (WHO, 2014). According to Rivers *et al*. (2014), the outbreak began in Guinea on March 23, 2014. The outbreak spread to yield wild spread and intense transmission in Guinea, Liberia and Sierra Leone, as well as cases in five additional countries. It is seen that arthropods, rodents and bats could be the host for Ebola virus (Olival *et al.*, 2013). Thus, the virus enters the human population through human contact with body parts or body fluids of a dead or living infectious animal (WHO, 2015). Thereafter, the disease then spreads within the human population through human to human mode of transmission (CDC, 2014).

Fever, headache, vomiting, watery diarrhea and abdominal pain are some of the symptoms of EVD which may appear anywhere between two to twenty one days after infection and at the time of no symptoms the individual is not infectious (CDC, 2014). It is possible for an EVD infected person to recover when the disease is effectively managed and controlled but immunity after recovery is not certain and the virus was found to be present in breast milk and semen even when it was no longer detectable in the patient’s blood. Further research showed that the virus was present in semen within the first 7 to 12 weeks after recovery from EVD. For these reasons, abstinence from both breastfeeding and unprotected sex is encouraged even after recovering from Ebola virus disease (WHO, 2015; Fisher *et al.*, 2016; Thorson *et al.*, 2016). So many EVD mathematical models with control measures have been developed and studied ever since the inception of the disease. Some models considered pharmaceutical control measures while others considered non pharmaceutical control measures. Using partial rank correlations and multivariate sensitivity analysis approach, Legrand *et al*. (2007) investigated the impact of isolation and barrier nursing control measures. Rivers *et al*. (2014) incorporated only pharmaceutical measures in their model while Madubueze *et al*. (2018) considered non pharmaceutical control measures such as quarantine and contact tracing. Rachah and Torres (2016) studied EVD dynamics using sensitivity analysis with the effect of vaccination in the population and Webb *et al*. (2015) implemented early identification and isolation of contact traced individuals that are infectious. We hereby consider both pharmaceutical and non-pharmaceutical control measures such as vaccine, treatment drugs, quarantine, isolation and condom use.

A study carried out in December, 2016 showed that the vaccine known as rVsv-EBOV is close to hundred percent effective in protecting against EVD infection, this was considered the first vaccine for the purpose of protection from the virus and is made available for use during outbreaks on compassionate ground.

During the 2018 Eastern Democratic Republic of the Congo outbreak, some treatment drugs were made available for EVD patients’ treatment of which regeneron (REGN-EB3) and mAb114 had higher overall survival outcome among the four drugs that were initially made available. These two drugs are currently in use for EVD patients and they work by stopping the virus from replicating itself (CDC, 2014).

At the time of the 2014 Ebola outbreak, when there was no vaccine or treatment drugs available for human use, quarantine and isolation were the control measures used to help reduce EVD spread in West Africa (Giubilini *et al.*, 2018). Quarantine is a control measure used in restricting the movement of those individuals that are exposed to a communicable disease such as EVD during the period of the virus incubation while isolation is used in separating the EVD symptomatic and infectious individuals from those that are healthy (Cetron *et al.*, 2004).

As a result of the virus being present in the semen of those that recovered from EVD and abstinence not easily implemented, condom use is a good control measure when the recovered individuals are released back into the population to help stem the disease spread.

This model is different from these other EVD mathematical models that have been used before now. It combines both pharmaceutical and non-pharmaceutical control measures and considers the susceptible vaccinated and unvaccinated condom users and non-condom users’ populations separately. The model provides possibilities for those exposed to the virus to take treatment drug at the onset.

This paper, using mathematical model approach formulated a model for EVD that combined vaccine, condom use, quarantine, isolation and treatment drug together as control measures in order to investigate the effect of combining these control measures in stopping EVD spread in human population.

## Materials and Methods

The paper has two distinguished population types viz: human and animal populations, each subdivided into mutually-exclusive compartments at time t. The total human population is denoted by; 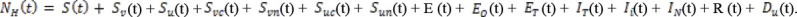.

The total animal population is denoted by 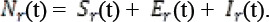


Thus, the total population is denoted by 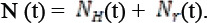


###  Assumptions about the model

- There is no herd immunity in the population.
- Isolated individuals are under close surveillance and do not contribute to the transmission of the infection.
- Vaccine, condoms, treatment drug, place of quarantine for the exposed and place for isolation for the infectious are all available and accessible to the population.
- The recovered individuals become susceptible to the virus again after some time.
- The infectious compartment is a transition point.
- The Ebola virus disease recovered individuals are still infectious through semen for some time.
- Ebola infected and infectious animals in the population have interactions with the susceptible human population.

### Model Variables’ Description

**S**: The susceptible population, **S_v_**: The susceptible vaccinated population, **S_u_**: The susceptible unvaccinated population, **S_vc_**: The susceptible vaccinated condom users population, **S_vn_**: The susceptible vaccinated non-condom users population, **S_uc_**: The susceptible unvaccinated condom users population, **S_un_**: The susceptible unvaccinated non-condom users population, **E**: The exposed population , **E_T_**: The exposed treated population, **E_Q_**: The exposed quarantined population, **I**: The infectious population, **I_i_**: The infectious isolated population, **I_T_**: The treated population, **I_N_**: The infectious not treated population, **R**: The recovered population, **D_u_**: The dead and unburied population, **S_r_**: The susceptible animal population, **E_r_**: The exposed animal population,

**I_r_**: The infectious animal population.

###  Model Equations

**Figure 1:**
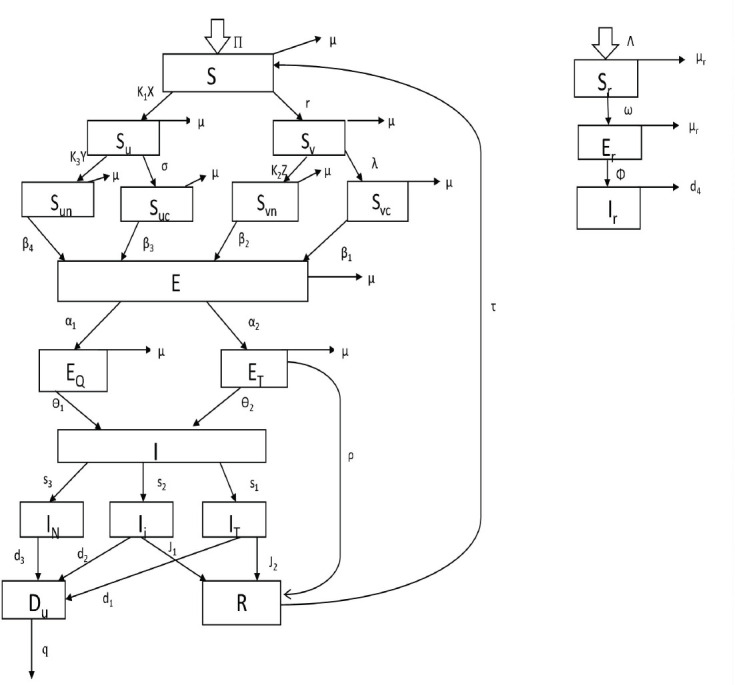
The Model Schematic Diagram


With our assumptions about the model and Figure 1, the following system of equations for our model was formulated;

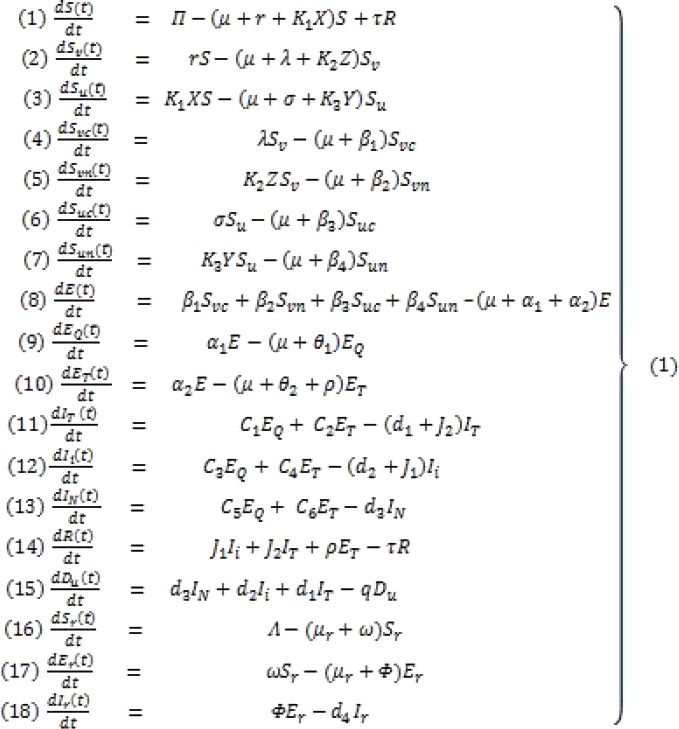


With the initial conditions given as follows:


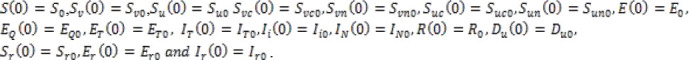


Where; d_1_= µ + ξ_1,_ d_2_ = µ + ξ_2_, d_3_= µ + ξ_3_, d_4_= µ + ξ_4_, X = (1-r), Z = (1-λ), Y = (1-σ), C_1_= ?_1_S_1_, C_2_= ?_2_S_1_, C_3_= ?_1_S_2_, C_4_=?_2_S_2_, C_5_ = ?_1_S_3_, C_6_= ?_2_S_3_ and


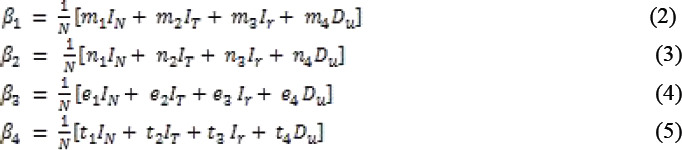


### Positivity of Solutions

Since the system of equations (1) represents human and animal populations. We must consider that the population size cannot be negative. Considering the biological feasible region

Ω = {(S,S_v_,S_u_,S_vc_,S_vn_,S_uc_,S_un_,E,E_Q_,E_T_,I_T_,I_i_,I_N_,R,D_u_,S_r_,E_r_,I_r_) ϵ 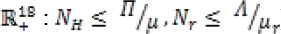


To ensure that all solutions in Ω remain in Ω for all time; whenever 
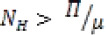
 then 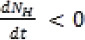 sand whenever 
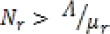
 then 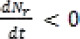 However, following that 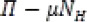 is a bound of 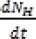 and 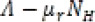 is a bound of 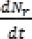 then

by recalling the implication of the comparison theorem, it can readily be shown that


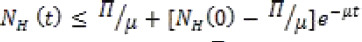
 and 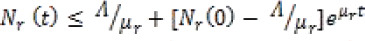


Obviously, 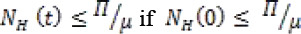 and 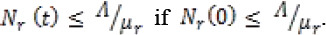


Hence, we conclude that every solution of the eighteen equations of the model (1) with initial conditions in Ω remains there for all t > 0. Thus, Ω is positively invariant and attracting. Therefore, it is sufficient to consider the dynamics of the flow generated by the system (1) in Ω. Thus, the model can be considered as being epidemiologically and mathematically well posed.

### Model Effective Reproduction Number (R_E_)

A better and widely used method in finding **R_E_** that reflects its biological meaning is the next generation operator method described by Dickmann and Heesterbeek (1990) and subsequently the method was analyzed by Van den Driessche and Watmough (2002). Using this method, we obtain the effective reproduction number for the system (1) which is the spectra radius ρ of the next generation matrix FV^-1^, that is **R_E_** = ρ(FV^-1^) and the spectral radius is the maximum eigenvalue of FV^-1^. Where F is a nonzero matrix that represents all new infection terms and V is an M-matrix representing all disease worsening terms. Considering the compartments E, E_Q_,E_T_,I_T_,I_i_,I_N_,R,D_u_,E_r_ and I_r_ we have the following;


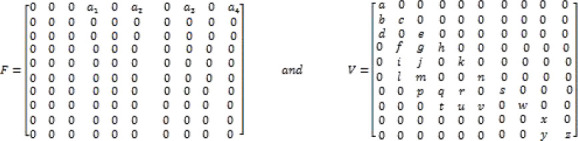


Using MATLab software, we obtained the effective reproduction number for our model (1) as;


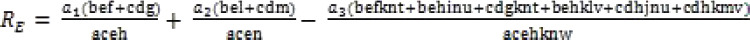


where; 
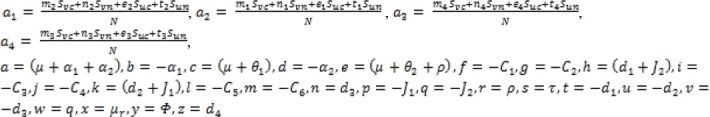


### The EVD - Free Equilibrium Point of the Model

The EVD - Free equilibrium is the state of total absence of Ebola virus disease in the entire population. At EVD - Free equilibrium state:


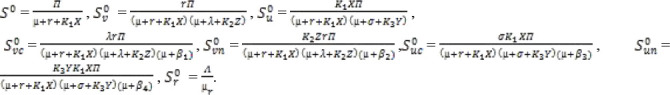


Thus, in the absence of the disease, the EVD - Free equilibrium of the model (1) exists and is given as;


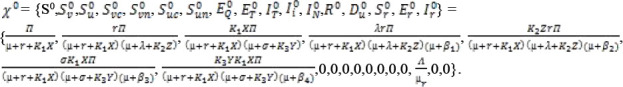


###  Local Stability of EVD – Free Equilibrium Point

At EVD -Free equilibrium; 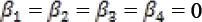


**Theorem 1:** The EVD -Free equilibrium point 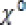 is locally asymptotically stable (LAS) if R_E_ < 1 and unstable if R_E_ > 1.

**Proof:** we employ the Jacobian stability technique of determining the local stability of a system such as (1). The Jacobian matrix of the system (1) at EVD -Free equilibrium 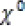
is given by;


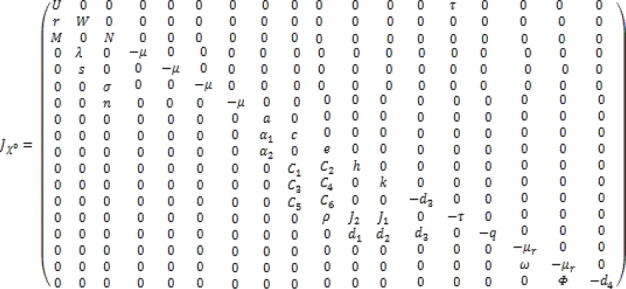


where; U = - (µ+ r + K_1_X), W = - (µ + λ + K_2_Z), N = - (µ + σ + K_3_Y), M = K_1_X, s = K_2_Z, n = K_3_Y,

a = - (µ + α_1_ + α_2_), c = - (µ + ?_1_), e = - (µ + ?_2_ + ρ), h = - (d_1_ + J_2_), k = - (d_2_ + J_1_).

Let the eigenvalues of the Jacobian matrix 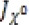 be η then 
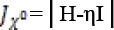
,

Evaluating we have the following corresponding eigenvalues; η_i_ where i = 1,2,3,…,16,17,18 and

η_1_ = - (µ + r + K_1_X) , η_2_ = - (µ + λ + K_2_Z) , η_3_ = - (µ + σ + K_3_Y) , η_4_ = - µ, η_5_ = - µ, η_6_ = - µ, η_7_ = - µ, η_8_ = - (µ + α_1_ + α_2_) , η_9_ = - (µ +?_1_), η_10_ = - (µ + ?_2_ + ρ) , η_11_ = -(d_1_ + J_2_) , η_12_ = - (d_2_ + J_1_) , η_13_ = - d_3_ , η_14_ = - τ , η_15_ = - q, η_11_ = - (d_1_ + J_2_), η_16_ = - µ_r_ , η_17_ = - µ_r_ , η_18_ = - d_4_.

Since all the eigenvalues have negative real parts, we then conclude that the EVD -Free equilibrium (DFE) is locally and asymptotically stable for 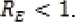.

###  The EVD - Endemic Equilibrium Point of the Model

To obtain the EVD – Endemic equilibrium point of the model we solve (1) simultaneously and have;


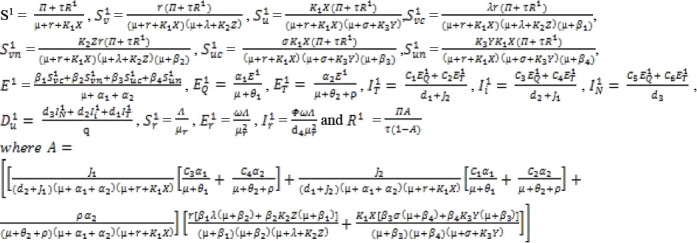


Thus, the EVD - Endemic equilibrium point of the model (1) exists and is given as


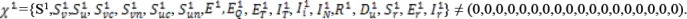


### Local Stability of the EVD - Endemic Equilibrium

EVD - Endemic equilibrium points are steady state solutions where all the state variables are positive. This means that EVD is present throughout in the population. The approach of center manifold theory described by Castillo - Chavez and Song (2004) used to investigate the stability of endemic equilibrium near 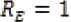 is applied. It is used to examine the existence of backward and forward bifurcation at 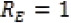. To achieve this, we make the following change of variables:

Let S = x_1_, S_v_ = x_2_, S_u_ = x_3_, S_vc_ = x_4_, S_vn_ = x_5_, S_uc_ = x_6_, S_un_ = x_7_, E = x_8_, E_Q_ = x_9_, E_T_ = x_10_, I_T_ = x_11_, I_i_ = x_12_, I_N_ = x_13_, R = x_14_, D_u_ = x_15_, S_r_ = x_16_,E_r_ = x_17_, I_r_ = x_18_.

Using the notation

X = (x_1_,x_2_,x_3_,…,x_17_,x_18_)^T^ ,

the model (1) can be expressed as


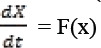


where F = (f_1_,f_2_,f_3_,…f_17_,f_18_)^T^ and


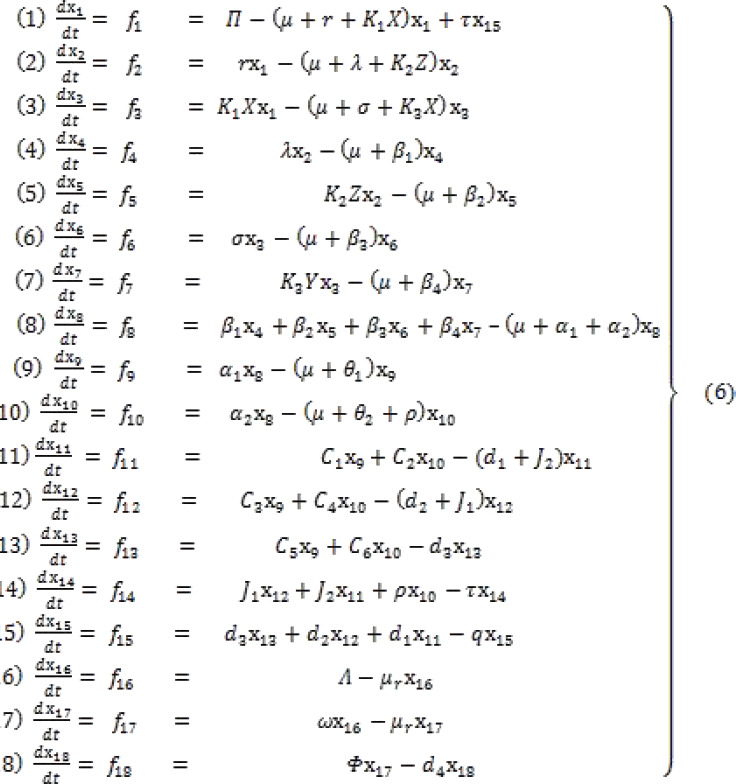


where;


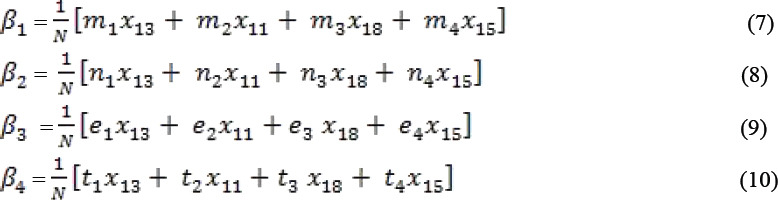


The Jacobian matrix associated with (6) at EVD - Endemic equilibrium is given as;


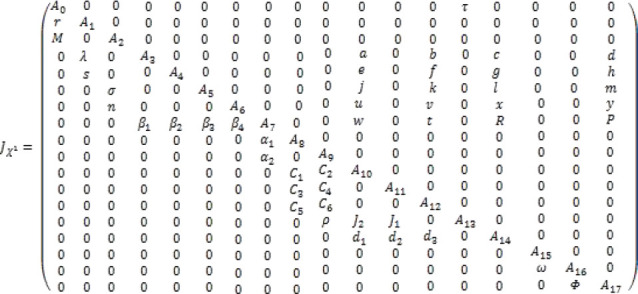


where; 
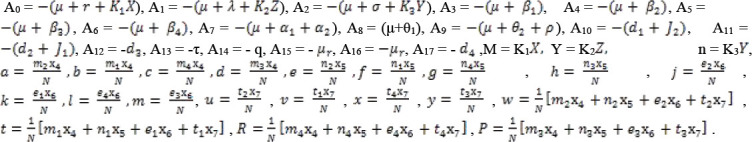


Considering 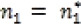 as our bifurcation parameter when 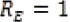 then;


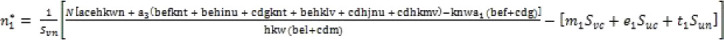


Supposing that the Jacobian matrix 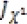 has V and W as its left and right eigenvectors respectively associated with its zero eigenvalues and are chosen in such a way that W.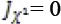 and V. 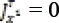 with V.W = 1, where

W = (w_1_, w_2_, w_3_, …, w_17_,w_18_)^T^ and V = (v_1_,v_2_,v_3_,…,v_17_,v_18_)^T^.

Then it follows that;


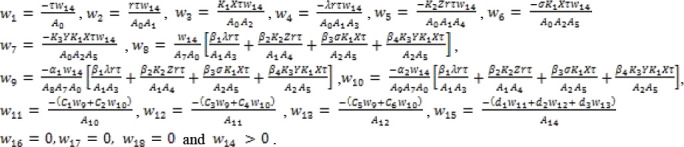


Similarly, 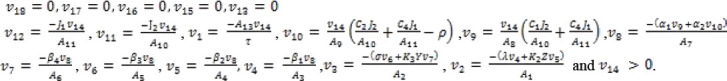


### Computations of a and b

With *n* = 18 and 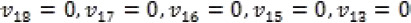, it follows that *a_18_ = a_17_ = a_16_ = a_15_ = a_13_ = 0* and all the partial derivatives of


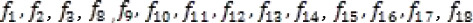
 are all zeros so we are left with the following;


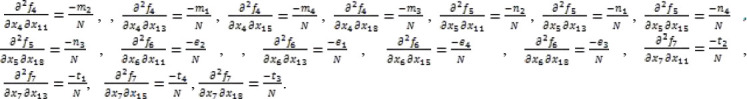



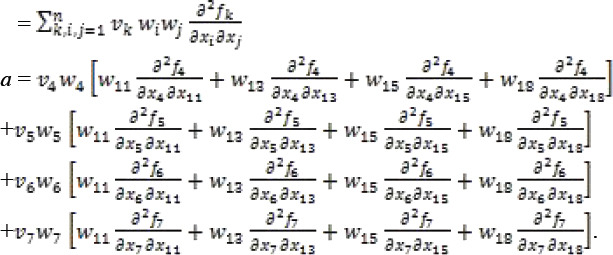


Note; 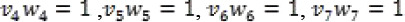 (since *v*.*w* = 1)

Thus,


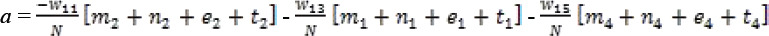
 (since 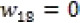 )

Therefore,

*a* < 0 (since 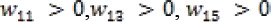 ).


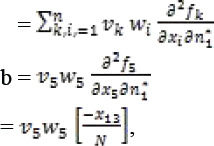


Thus, b < 0 (Since 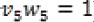).

Since *a* < 0 and *b* < 0

*we* then conclude that the EVD - Endemic equilibrium of the model (1) is locally asymptotically stable for *R_E_* > 1 and close to 1 and exhibits a forward bifurcation at *R_E_* = 1.

### Numerical Simulations

Using the computer software known as MATLab we perform numerical simulations on our model (1) in order to see the effect of all the control measures incorporated into the model (1).The model(1) was implemented in MATLab and was parameterized for the Liberian situation during the 2014 Ebola outbreak. The population of Liberia was estimated to be 4396554 in the year 2014. We then estimate our initial values and parameters as follows; S (0) = 4396521, I (0) = 33, D_u_ (0) = 24, E_Q_ (0)=74 (Madubueze *et al.*, 2018), E (0) = 0, E_T_ (0) = 0,I_i_ (0) = 33 – 24 =9, I_T_ (0) = 0, I_N_ (0) = 0, R (0) = 0,S_v_ (0) = 1758608, S_u_ (0) = 2637913, S_vc_ = 527582, S_vn_ (0) = 1231026, S_uc_ (0) = 1582748, S_un_ (0) = 1055165,S_r_ (0) = 6000, E_r_ (0) = 0, I_r_ (0) = 0.

## Results

With the initial values and parameter values in Table 1 the following results were obtained from MATLab;

**Table 1:** Model Parameters’ Description and Values

Parameters	Description	Values	Source
**Π**	Susceptible human population recruitment rate	**422**	**Madubueze *et al*., (2018)**
**μ**	Human natural death rate	**0.0000246575**	**Madubueze *et al*., (2018)**
**r**	Susceptible population’s rate of vaccination	**0.05**	**Estimated**
	Susceptible vaccinated Population’s rate of using condom	**0.002**	**Estimated**
**σ**	Susceptible unvaccinated Population’s rate of using condom	**0.07**	**Estimated**
**α_1_**	Exposed population’s rate of quarantine	**0.07143**	**Gomes *et al*., (2014)**
**α_2_**	Exposed population’s rate of treatment	**0.02741**	**Estimated**
**θ_1_**	Exposed quarantined population’s rate of becoming infectious	**0.08333**	**Legrand *et al*., (2007)**
**θ_2_**	Exposed treated population’s rate of becoming infectious	**0.014**	**Estimated**
**s_1_**	Rate of treatment for the infectious	**0.2257**	**Gomes *et al*., (2014)**
**s_2_**	Rate of isolation for the infectious	**0.25**	**WHO (2014)**
**s_3_**	Rate of no treatment for the infectious	**0.148**	**Estimated**
**K_1_**	Probability constant that Su remains unvaccinated	**0.895**	**Estimated**
**K_2_**	Probability constant that Svn remain not using condom	**0.01**	**Estimated**
**K_3_**	Probability constant that Sun remains not using condom	**0.002**	**Estimated**
**ξ_1_**	Disease induced death rate for the infectious treated population	**0.11386**	**Gomes *et al*., (2014)**
**ξ_2_**	Disease induced death rate for the infectious isolated population	**0.0901**	**Rivers *et al*., (2014)**
**ξ_3_**	Disease induced death rate for the infectious not treated population	**0.2443**	**Estimated**
**ξ_4_**	Disease induced death rate for the infectious animal population	**0.3110**	**Estimated**
**β_1_**	Rate of infection for Svc	**0.1**	**Estimated**
**β_2_**	Rate of infection for Svn	**0.2**	**Estimated**
**β_3_**	Rate of infection for Suc	**0.3**	**Estimated**
**β_4_**	Rate of infection for Sun	**0.4**	**Estimated**
**Λ**	Susceptible animal population recruitment rate	**100**	**Estimated**
**ω**	Susceptible animal population rate of exposure	**0.5**	**Estimated**
**J_1_**	Rate of recovery for the infectious treated individuals	**0.105186**	**Gomes *et al*. , (2014)**
**J_2_**	Rate of recovery for the infectious isolated individuals	**0.17**	**Rivers *et al*. , (2014)**
**ρ**	Rate at which the recovered become susceptible	**0.06**	**Rivers *et al*. , (2014)**
**ρ**	Rate of recovery for the exposed treated individuals	**0.0314862**	**Rivers *et al*. , (2014)**
**Φ**	Rate at which exposed animals become infectious	**0.6**	**Estimated**
**μ_r_**	Animal population natural death rate	**0.08**	**Estimated**
**q**	Rate of burial for the dead and unburied population	**0.5**	**Estimated**

**Figure 2:**
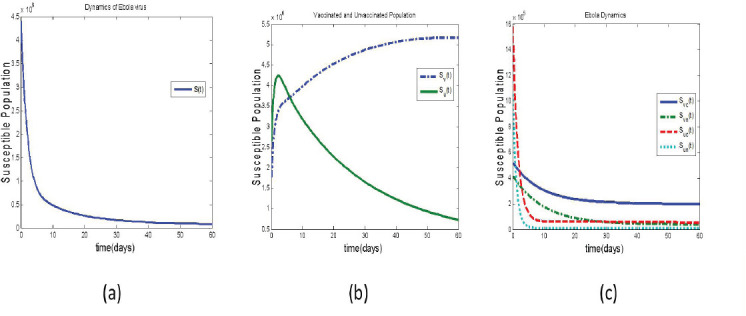
The Graph of the Susceptible Human Populations

**Figure 2**: The simulation graph showed the graphs of the human susceptible populations (S, S_v_, S_u_, S_vc_, S_vn_, S_uc_ and S_un_) over a period of 60 days. It showed that the susceptible population decreased with time but never got to zero because more people are being recruited into the population. The vaccinated and unvaccinated populations both increased initially, and the unvaccinated population reduced drastically with time because of no vaccine implementation in the population. The susceptible vaccinated and unvaccinated condom users and non-condom users populations all decreased with time but the susceptible unvaccinated non condom users population decreased the most and faster also while the susceptible vaccinated condom users population decreased the least and slower because of vaccine and condom use implementation in the population.

This implies that with the implementation of vaccine and condom use in the susceptible population, less people will be exposed to the virus at a slower rate.

**Figure 3:**
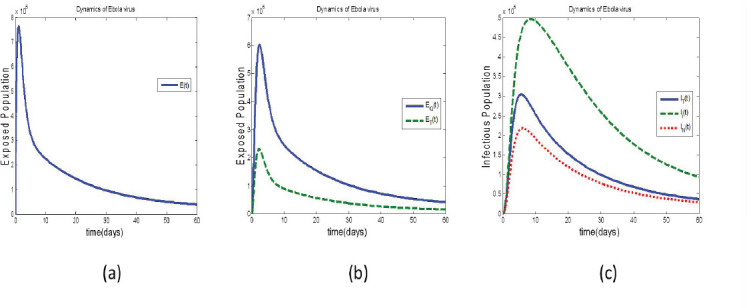
The Graph of the Exposed and Infectious Human Populations

**Figure 3:** The simulation graphs showed the graphs of the human exposed and infectious populations (E, E_Q_, E_T_, I_T_, I_i_ and I_N_) over a period of 60 days. it showed that the exposed population drastically increased from initial time because of human fast exposure to the virus initially and got to its peak before 10 days before the population reduced with time. The exposed treated and quarantined populations both increased also before they decreased but the exposed quarantined population increased the most because more exposed people are going for quarantine in the population. The infectious populations also increased and later decreased with time but the infectious isolated population increased the most while the infectious not treated population increased the least because more infectious people are going for isolation and few infectious people are going for treatment drug.

This implies that among the exposed population more people are going for quarantine than treatment drugs. Also, among the infectious population, more people are going for isolation and lesser people are going without any kind of treatment.

**Figure 4:**
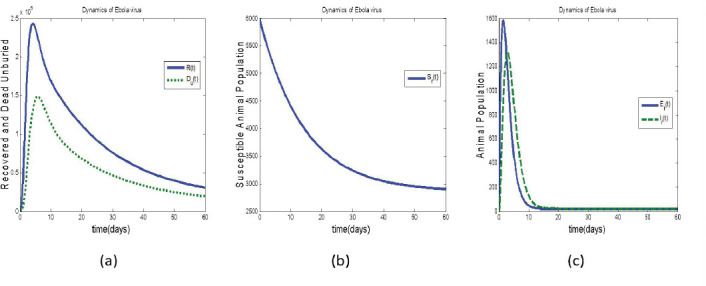
The Graph of the Recovered, Dead and Unburied and the Animal Populations

**Figure 4**: The simulation graphs showed the human recovered and dead and unburied populations together with the animal populations (R, D_u_, S_r_, E_r_ and I_r_) over a period of 60 days. it showed that the recovered and dead and unburied populations both increased initially, got to their peaks and decrease with time but the recovered population increased the most as more people are recovering from EVD and less people are dying from EVD with the combined implementation of our control measures. The susceptible animal population decreased with time but did not get to zero as more animals are being recruited into the animal population. Also, the exposed and infectious animal populations both increased and got to their peaks before they both decreased but the exposed animal population increased the most as some exposed animals died before becoming infectious.

This implies that with all the control measures implemented more people will recover from the disease, while less people will die from the disease.

## Discussion

In this study, the general epidemiology of the Ebola Virus Disease is considered, and we formulated a simulation model for the dynamics of Ebola Virus Disease detailed with recent developments in the area of the disease. This is to help in preventing outbreaks which might be fatal in the future. We used the simulation model formulated in this study for the spread of the virus in Liberia and to project how an epidemic might go under the implementation of all our incorporated control measures together in the area. Liberia information and data from World Health Organization during the 2014 outbreak in West Africa was used in this study. In our model, we assumed that the interventions incorporated are not in any way hindered by any lack of resources. The positivity of solution for the model showed that the epidemic system possess non negative solutions under a non-negative initial conditions. This means that the population considered with all its sub populations cannot be negative. The effective reproduction number R_E_ for the model was computed and used in the stability analysis of the equilibrium points of our model.

The EVD Free equilibrium of the model was computed and found to be locally asymptotically stable as long as R_E_ < 1, which means that the disease would be eliminated from the system within a short period of time when our control measures are well implemented together. The EVD Endemic equilibrium was also computed and found to be locally stable when R_E_ > 1 and exhibits a forward bifurcation when R_E_ = 1. This means that the control of the virus in the population is independent on the number of the individuals initially infected. The disease will be eradicated from the population when R_E_ < 1 and the disease will not be eradicated in the population when R_E_ > 1.

Since the bifurcation is forward, global stability for the model will also exist because it is in backward bifurcation that global stability may not exist. Finally, the numerical simulation of our model was done and the summary of the obtained results are as follows;

**Figure 2: (a)** The graph of the susceptible human population is decreasing and does not get to zero with time. This decrease is because people are getting vaccinated and leaving the population. The population does not get to zero as people are recruited into the population and the recovered individuals rejoin the population. **(b)** The graph of the susceptible vaccinated and unvaccinated human populations shows an initial sharp increase and a drastic decrease with time for the unvaccinated population while there is a gradual and steady kind of increase for the vaccinated population. This is because initially people where not going for the vaccine but with time more people began to go for the vaccine as more unvaccinated individuals where getting infected.

**(c)** The graph of the susceptible vaccinated (condom users and non condom users) and unvaccinated (condom users and non condom users) human populations shows that the unvaccinated non condom users population decreased the most followed by the unvaccinated condom users population, followed by the vaccinated non condom users population while the vaccinated condom users population decreased the least because of the implementation of vaccine with condom use in the population.

This shows that when there is no implementation of vaccine and condom use in an EVD susceptible population many people will be exposed to the virus drastically, when only condom use is implemented in an unvaccinated EVD susceptible population many people will still be drastically exposed to the virus. When there is implementation of vaccine without condom use in an EVD susceptible population lesser number of people will become exposed to the virus gradually but when condom use is implemented in an EVD susceptible vaccinated population a very minimal number of people get exposed to the virus at a slower rate. Thus, the best strategy for an EVD susceptible population is the combination of vaccine together with condom use.

**Figure 3**: **(a)** The graph of the exposed human population shows a sharp increase from the initial time because of no control measure in place initially in the population, on getting to its peak it began to decrease as a result of the implementation of quarantine and treatment drug in the exposed population.

**(b)** The graph of the exposed quarantined human population with the exposed treated population shows initial increase for both population as a result of those exposed to the virus going into quarantine and others going for treatment drugs but those going for quarantine are more than those going for treatment drugs and both population begin to reduce with time as a result of some becoming infectious and others recovering.

**(c)** The graph of the infectious (treated, isolated and not treated) human population shows each of them initially drastically increasing and later began to decrease with time. The graph shows that more infectious people go for isolation and less infectious people are without any form of treatment. The infectious treated and isolated populations decrease as a result of some recovering and others die from the disease. The infectious not treated population decrease as a result of death from the disease.

This shows that if the treatment drug for EVD is made affordable to all in such a way that those exposed to the disease can buy and take when exposed then much exposed people can recover without becoming infectious and even those infectious can take the drug and have a better chance at recovering from the disease. Also the implementation of isolation for the infectious will go a long way in drastically reducing the disease spread.

**Figure 4**: **(a)** The graph of the recovered and dead unburied human populations shows both populations increasing from initial time. They both begin to reduce gradually with time as the recovered goes back to being susceptible again and the dead unburied are buried with time.

**(b)** The graph of the susceptible animal population shows the population reducing with time and not getting to zero. This is because the susceptible animals are being exposed to the virus while more animals are being recruited into the population with time.

**(c)** The graph of the exposed and infectious animal populations shows that the exposed animal population increased as more animals were getting infected and began reduced as they became infectious while the infectious animal population increased as more exposed animals became infectious and reduced as some of the infectious animals began to die off.

This shows that the number of recovered human is far more than the number of EVD dead human in the population. This implies that with the implementation of vaccine, condom use, quarantine, isolation and treatment drug more people will recover from the disease while lesser people will die from the disease even when some are exposed.

Thus, our graph confirmed our analysis as it showed that the disease can be controlled and eradicated with time in the population irrespective of the initial number of infected seeing that all our infected and infectious populations are almost zero as at 60 days. Therefore, when all our control measures are incorporated together and used well the population will not experience a serious and prolonged EVD outbreak.

## Conclusion

In this work, we formulated a classical model for the dynamics of Ebola Virus Disease and the model was analyzed. The effective reproduction number for the model R_E_ was obtained using the next generation matrix. The existence of the EVD Free equilibrium was established and shown to be locally asymptotically stable when R_E_ < 1 using the Jacobian matrix technique. The EVD Endemic equilibrium existence was also established and found to be locally asymptotically stable when R_E_ > 1 and exhibits a forward bifurcation at R_E_ = 1 using center manifold theorem.

Finally, the numerical simulation of the model was carried out using MATLab computer software to examine the effect of the combination of all our incorporated control measures on the transmission dynamics of the disease. The result showed that the combined implementation of vaccine, condom use, quarantine, isolation and treatment drugs measures has great significance in effectively controlling Ebola Virus Disease in the population as it helped bring down the disease spread in the population within 60 days.

**List of Abbreviations:**
(EVD): Ebola Virus Disease
(EBOV): Zaire Ebola Virus
(WHO): World Health Organization
